# Disulfonated Poly(arylene ether sulfone) Random Copolymers Containing Hierarchical Iptycene Units for Proton Exchange Membranes

**DOI:** 10.3389/fchem.2020.00674

**Published:** 2020-08-04

**Authors:** Tao Wang, Tianyun Li, Joseph Aboki, Ruilan Guo

**Affiliations:** Department of Chemical and Biomolecular Engineering, University of Notre Dame, Notre Dame, IN, United States

**Keywords:** iptycene-containing poly(arylene ether sulfone), random copolymers, high free volume, proton exchange membrane, fuel cell

## Abstract

Two series of disulfonated iptycene-based poly(arylene ether sulfone) random copolymers, i.e., TRP-BP (triptycene-based) and PENT-BP (pentiptycene-based), were synthesized via condensation polymerization from disulfonated monomer and comonomers to prepare proton exchange membranes (PEMs) for potential applications in electrochemical devices such as fuel cell. To investigate the effect of iptycene units on membrane performance, these copolymers were systematically varied in composition (i.e., iptycene content) and the degree of sulfonation (i.e., 30–50%), which were characterized comprehensively in terms of water uptake, swelling ratio, oxidative stability, thermal and mechanical properties, and proton conductivity at various temperatures. Comparing to copolymers without iptycene units, TRP-BP and PENT-BP ionomers showed greatly enhanced thermal and oxidative stabilities due to strong intra- and inter-molecular supramolecular interactions induced by hierarchical iptycene units. In addition, the introduction of iptycene units in general provides PEMs with exceptional dimensional stability of low volume swelling ratio at high water uptakes, which is ascribed to the supramolecularly interlocked structure as well as high fractional free volume of iptycene-based polymers. It is demonstrated that the combination of high proton conductivity and good membrane dimension stability is the result of the synergistic effects of multiple factors including free volume (iptycene content), sulfonation degree, hydrophobicity, and swelling behavior (supramolecular interactions).

## Introduction

With the increasing world population and limited fossil energy sources, clean, and sustainable energy has become a hot topic (Dincer, 2000; Omer, 2008; Chu and Majumdar, 2012). Fuel cells are regarded as promising energy devices for vehicle propulsion, stationary, and portable power with high energy conversion, decent energy density, and sustainability (Hickner et al., 2004; Smitha et al., 2005; Peighambardoust et al., 2010; Wang et al., 2011). Proton exchange membrane fuel cells (PEMFCs) using proton exchange membranes (PEMs) as solid electrolyte are widely studied for their high proton conductivity, good chemical, thermal, and mechanical stabilities. The state-of-the-art proton exchange membrane Nafion® produced by Dupont has dominated the commercial field for their exceptional chemical and mechanical stabilities as well as good conductivities, resulting from the poly(perfluorosulfonic acid) structure. However, the drawbacks of high cost, low operation temperature, poor conductivity at low relative humidity, and high fuel permeability promote the research for alternative non-fluorinated structures such as poly(arylene ether)s, poly(imide)s and poly(ether sulfone)s (Ghassemi and McGrath, 2004; Harrison et al., 2005; Liu et al., 2007; Miyatake et al., 2007; Matsumoto et al., 2008). Among the diverse structures reported in the literature, disulfonated poly(arylene ether sulfone) copolymers, such as BPSH series (Wang et al., 2002) are promising candidates for PEMFCs for their high proton conductivities with facile synthesis. Much efforts have been focused on modifying the membranes, including but not limited to introducing pendant groups (Wang et al., 2012; Zhao et al., 2013; Zhou et al., 2020), developing acid-base membranes (Yue et al., 2017; Ahn et al., 2018), preparing block copolymers (Lee et al., 2008; Li et al., 2012; Assumma et al., 2015), and blending sulfonated polysulfone with other polymers and fillers (Bi et al., 2010; Liang et al., 2013; Wang et al., 2019). Nevertheless, there is still a lot of room to improve for the polysulfone-based PEMs in terms of proton conductivities and dimensional stabilities to enable practical applications.

Recently, incorporating bulky, space-occupying (i.e., high free volume) moieties into ionic polymers has attracted a lot of attention for various membrane applications including PEMFCs. The main idea is to enable high water uptake via high fractional free volume to address the loss of water at low humidity and high temperature while suppressing excessive water swelling via highly rigid backbone structure. Zhang and his co-workers first introduced poly(arylene ether sulfone) random copolymers containing iptycene (i.e., triptycene and pentiptycene) with post-sulfonation treatment and reported reduced water swelling without loss of proton conductivities (Gong and Zhang, 2011; Gong et al., 2011). Swager group recently found that triptycene-based poly(ether ketone)s with increased fractional free volume showed enhanced proton conductivities under the conditions of low relative humidity and high temperature (Moh et al., 2018). They attributed the enhanced performance to the strong ability of retaining more water molecules and relieving the high internal energy enabled by high intrinsic free volume associated with bulky triptycene moieties in the polymer structures.

However, most existing studies of ionic polymers containing bulky structure units used post-polymerization sulfonation methods to introduce ionic groups (e.g., sulfonate) into polymer structures, where aromatic polymers were sulfonated by concentrated sulfuric acid, chlorosulfonic acid, fuming sulfuric acid, or sulfurtrioxide. Post-polymerization sulfonation in general is not able to attain precise control of the sulfonation degree and the location or distribution of ionic groups, and usually has low sulfonation efficiency. Moreover, post modifications are frequently accompanied with possible polymer backbone degradation and undesired side reactions due to the use of strong acidic media that result in deteriorated membrane properties (Hickner et al., 2004). For example, Genova-Dimitrova et al. (2001) reported that the use of strong sulfonating agent induced chain cleavage, as evidenced by the decreased viscosity upon post sulfonation. In this regard, preparing ion-containing polymers directly from disulfonated ionic monomers is beneficial in terms of better controlling the sulfonation degree and the location of sulfonate groups and achieving high molecular weight in resulting ionic polymers. Recently, we reported triptycene-containing poly(arylene ether sulfone) multiblock copolymers prepared directly from sulfonated monomers, which exhibit excellent dimensional stability with low swelling ratios despite of their relatively high water uptake (Aboki et al., 2018). We demonstrated that the supramolecular interlocking interactions and π-π stacking associated with triptycene units in the hydrophobic blocks are instrumental in suppressing the undesired excessive water-swelling behavior frequently observed in common ionic membranes. The excellent properties of triptycene-containing PEMs and related ionic exchange membranes with high free volume motivate us to further explore the potential of high-free-volume ionic polymers, in particular, sulfonated poly(arylene ether sulfone)s for PEMFCs applications.

In this work, we report the use of iptycene-based (i.e., triptycene and pentiptycene) structure units and sulfonated monomer to prepare disulfonated poly(arylene ether sulfone) random copolymers with systematically varied composition and functionality. The extension to pentiptycene moieties, which has even bulkier structure and higher intrinsic internal free volume than triptycene ones (Luo et al., 2015), is expected to retain more water molecules and facilitate the proton conductivity at low relative humidity (RH) and high temperature. Moreover, with more arene rings participating in strong supramolecular interactions and π-π stacking, more rigid scaffolds are expected to form in pentiptycene-based structures that may further improve mechanical and dimensional stabilities. Specifically, two series of iptycene-containing, sulfonated polysulfone random copolymers are prepared via direct polycondensation reactions and comprehensively examined for PEMFC applications, including triptycene-based TRP-BP series and pentiptycene-based PENT-BP series with systematically varied iptycene content and sulfonation degree. Direct comparisons with non-iptycene-containing BPSH series are made to elucidate the effect of the incorporation of triptycene or pentiptycene moieties on PEM properties. Comprehensive investigations of physical properties, chemical structure, proton conductivity, and thermal, mechanical, and oxidative stabilities of these new iptycene-based sulfonated copolymers were conducted to establish the fundamental structure-property relationships for these new PEM materials, which would serve as a guide to new designs of high-performance PEMs.

## Experimental

### Materials

Triptycene-1,4-diol (TRP) (Wiegand et al., 2014) and pentiptycene-6,13-diol (PENT) (Luo et al., 2015) were synthesized according to previous literature. Anthracene, sodium hydrosulfite, 4,4′-dichlorodiphenylsulfone (DCDPS), and 4,4′-difluorodiphenylsulfone (DFDPS) were purchased from Alfa Aesar and used as received. 1,4-benzoquinone, tetrachloro-1,4-benzoquinone, potassium carbonate (K_2_CO_3_), sodium bicarbonate, acetic acid, methanol, and 2-propanol were purchased from Sigma-Aldrich. 4,4′-biphenol (BP) and 3,3′-disulfonated-4,4′-dichlorodiphenylsulfone (SDCDPS) were purchased from Akron Polymer Systems and dried in vacuum at 110°C for 24 h before use. Anhydrous *N, N*-dimethylacetamide (DMAc), toluene, and hydrogen peroxide (30 wt% solution) were purchased from EMD Millipore and used as received.

### Synthesis of Disulfonated Iptycene-Containing Poly(Arylene Ether Sulfone) Copolymers

#### Triptycene-Containing Copolymers

**Scheme 1:**
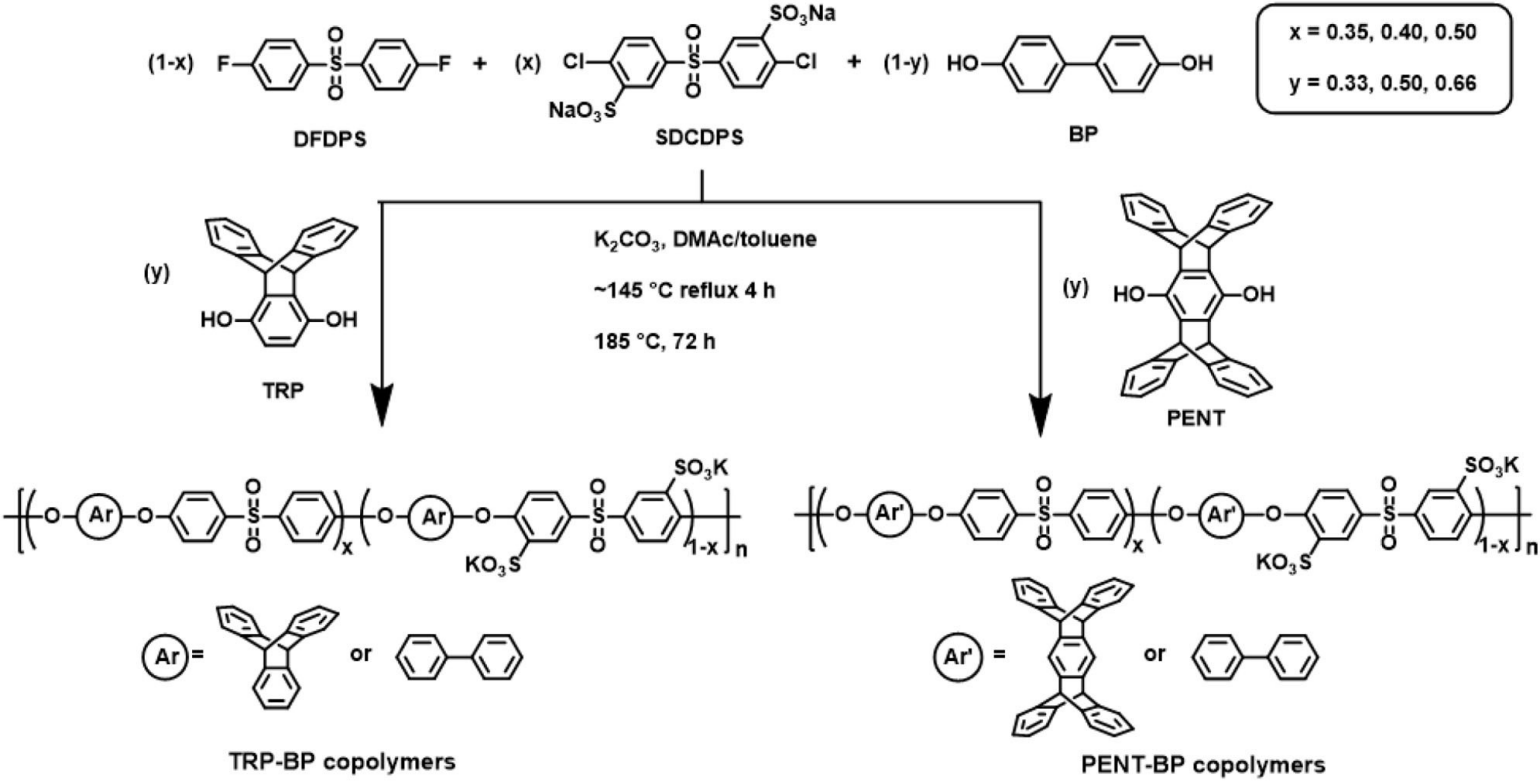
Synthesis of TRP-BP and PENT-BP disulfonated random copolymers with controlled composition.

A series of triptycene-containing disulfonated poly(arylene ether sulfone) random copolymers (i.e., TRP-BP series) with different molar ratios of TRP to BP and varied degrees of sulfonation were synthesized via nucleophilic polycondensation according to reported method (Scheme 1) (Luo et al., 2018). The nomenclature used for the copolymers is TRP-BP a:b-X, where a:b is the TRP:BP molar ratio and X is the molar percentage of sulfonated SDCDPS relative to non-sulfonated DCDPS in the copolymers, as shown in Scheme 1. For example, TRP-BP 1:1-35 refers to the copolymer containing 1:1 molar ratio of TRP:BP, 35 mol% sulfonated monomer (SDCDPS), and 65 mol% non-sulfonated monomer (DCDPS). It should be noted that random copolymers with high TRP molar contents (>66 mol%) and high degree of sulfonation (>50 mol%) did not have sufficiently high molecular weight for film formation due to the low reactivity between TRP and SDCDPS. A sample polymerization of TRP-BP 1:1-35 is as follows: 1.8621 g (10.0 mmol) of BP, 2.8633 g (10.0 mmol) of TRP, 3.4386 g (7.0 mmol) of SDCDPS, 3.7330 g (13.0 mmol) of DCDPS, and 3.3169 g (24.0 mmol) of anhydrous K_2_CO_3_ were charged into a three-necked round-bottom flask equipped with a condenser, mechanical stirrer, Dean-Stark trap, and nitrogen inlet. Anhydrous DMAc (60 mL) and toluene (30 mL) were then added to the flask, and the reaction was heated, under a N_2_ purge, to 145°C while stirring. The reaction was refluxed at 145°C for 4 h to azeotropically dehydrate the system. Afterward, toluene was removed from the reaction by slowly increasing the temperature to 185°C. The reaction was allowed to proceed at 185°C for another 72 h until a viscous solution formed. The polymer solution was then cooled to room temperature, vacuum filtered to remove salts, and coagulated in a stirred 2-propanol bath. The precipitated fibrous TRP-BP copolymer was collected and dried under vacuum at 120°C for 24 h.

#### Pentiptycene-Containing Copolymers

Similar procedures were taken to synthesize pentiptycene-containing disulfonated polysulfone random copolymer series, i.e., PENT-BP series, with varied pentiptycene content and degree of sulfonation. The same naming system is applied to the PENT-BP series. A typical synthesis of PENT-BP 1:2-40 (1:2 refers to the molar ratio of PENT:BP and 40 is the sulfonation degree of the copolymer) is described as follows: 2.3127 g PENT (5 mmol), 1.8621 g BP (10 mmol), 2.9474 g SDCDPS (6 mmol), 2.2883 g DFDPS (9 mmol) and 4.1462 g K_2_CO_3_ (30 mmol) were charged into a 100 mL three-neck flask with a nitrogen inlet, a mechanical stirrer, and a Dean-Stark trap. Then 50 mL anhydrous DMAc and 25 mL toluene were added and the reaction mixture was refluxed at 145°C for 4 h to azeotropically dehydrate the system. While removing the toluene, the temperature was slowly increased to 185°C and hold for 72 h. The resulting viscous polymer solution was filtered to remove salts and then precipitated in methanol with stirring. The fibrous copolymer was then collected and dried in vacuum oven for 24 h at 120°C.

As a control for comparisons to investigate the effect of iptycene structure, a series of non-iptycene-containing BPSH random copolymers with controlled degree of sulfonation were also synthesized from BP, DCDPS, and SDCDPS according to previous reports (Wang et al., 2001, 2002).

### Film Preparation and Acidification

All salt-form (as synthesized) copolymer films were prepared via a solution casting method. In a typical case, the salt-form copolymers were dissolved in DMAc to form a ~7% w/v solution, which was filtered through 0.45 μm Teflon® syringe filters. The filtered solutions were cast onto clean, leveled glass plates and dried under an infrared lamp at ~55°C for 24 h to form polymer films. The residual solvent was further removed by drying the film under vacuum at 120°C for 24 h.

The as-cast salt-form thin films were converted to acid form—that is, K^+^ was replaced with H^+^ in the copolymer structure shown in Scheme 1—by boiling the films in 0.5 M sulfuric acid solution for 2 h followed by boiling the films in deionized (DI) water for another 2 h (Kim et al., 2004). All obtained acid-form polymer films with thickness between 30 and 70 μm were stored in DI water until use.

### Characterization of Polymers and Films

Intrinsic viscosity (IV) of salt-form copolymers was determined in 0.05 M LiBr-NMP solution at 25°C using a Cannon-Ubbelohde viscometer and the average value of at least three measurements was reported. The density of dry acid-form membrane was measured using a density measurement kit (ML-DNY-43, Mettler Toledo) and an analytical balance (ML204, Metter Toledo) at room temperature. The dry membrane density ρ_*dry*_, was determined according to Archimedes' principle as follows:

(1) $$\begin{array}{l} \rho_{dry}=\frac{m_{air}}{m_{air}-m_{wet}}\left(\rho_{aux}-\rho_{air}\right) \end{array}$$

where *m*_*air*_ is the membrane weight measured in air and *m*_*wet*_ is measured in cyclohexane. The density of cyclohexane ρ_*aux*_ was determined at measured temperature and ρ_*air*_ was taken as 0.0012 g/cm^3^ (Luo et al., 2018).

The thermal stability (weight loss) of the acid-form membranes was evaluated by thermogravimetric analysis (TGA) using a TA instrument Q500. The samples were heated from 50 to 800°C at a heating rate of 10°C/min under N_2_ atmosphere after drying at 150°C for 30 min in the TGA furnace. The differential scanning calorimetry (DSC) analyses were done on a TA instrument Q2000 with liquid nitrogen cooling system at a heating rate of 10°C/min. Prior to the measurement, membranes were dried under vacuum at 120°C for 24 h to fully remove residual solvents. The samples were tested within the range of 50–325°C and the glass transition temperature (*T*_*g*_) was obtained from the second heating cycle (Aboki et al., 2018).

Membrane oxidative stability was tested by immersing the films in hot Fenton's reagent (3% H_2_O_2_ aqueous solution containing 2 ppm FeSO_4_) at 80°C for 1 h. Then the residual weight (*RW*) was measured after drying the remaining film in vacuum oven at 100°C overnight.

The dry-weight ion exchange capacity (IEC_w_, meq/g) (Hickner et al., 2004) of all acid-form membranes was determined by acid-base titration method. Dry membranes were immersed in 5 M NaCl solution for 48 h to release the H^+^. Then the solution was titrated by 0.01 M NaOH solution using phenolphthalein as indicator. The dry-weight-based IEC_w_ was calculated as follows,

(2) $$\begin{array}{l} IEC_{w}\left(meq/g\right)=\frac{c_{NaOH}\;\times V_{NaOH}\;}{m\;} \end{array}$$

where *c*_*NaOH*_ and *V*_*NaOH*_ are the concentration and the used volume of NaOH solution, respectively, and *m* is the mass of the sample. Average values of IEC_w_ were obtained and reported from at least 3 samples for each film.

Mechanical properties of the acid-form films were determined via uniaxial tensile testing with a Bose Electro-Force 3300 instrument with 25 lb load cell at 25°C following our previous method (Aboki et al., 2018). The hydrated specimens were prepared as dumbbell shape specimens of 25 × 10 mm (total) and 10 × 5 mm (test area). The stress-strain curve was obtained at a stretching rate of 2 mm/min. At least three samples were tested for each copolymer and the average value was reported.

Water uptake (WU) of acid-form membranes was evaluated by immersing fully dried polymer films (*W*_*dry*_) in DI water at room temperature for 24 h. The membranes were wiped using Kimwipe® to remove any surface water and quickly weighed (*W*_*wet*_). WU in weight percent was calculated as follows:

(3) $$\begin{array}{l} WU\;\left(wt\%\right)=\frac{W_{wet}-W_{dry}}{W_{dry}}\times 100\% \end{array}$$

Swelling ratio (SR) of the membranes was determined by measuring the volume change after immersing the films in DI water. The acid-form membranes were cut into rectangular shape and immersed in DI water at room temperature for 24 h. The dimension (i.e., length, width, and thickness) of the films were measured before and after water immersion to calculate the dry and wet volume of the films. Then the *SR* was calculated by the following equation:

(4) $$\begin{array}{l} SR\left(\%\right)=\frac{V_{wet}-V_{dry}}{V_{dry}}\times 100\% \end{array}$$

To account for membrane swelling, the wet volume-based IEC values were also calculated from the weight-based IEC_w_, density and swelling ratio as follows,

(5) $$\begin{array}{l} IEC_{v}\left(wet\right)\left(meq/cm^{3}\right)=\frac{IEC_{w}\times \rho \;}{\left(1+SR\right)} \end{array}$$

where *IEC*_*w*_, density and swelling ratio (SR) are all from experimentally determined values.

### Proton Conductivity Measurement

The proton conductivity of the acid-form films under fully hydrated condition was measured in DI water on a PARSTAT® MC 1,000 impedance spectrometer via Linear Sweep Voltammeter (LSV) using a 4-electrode BekkTech Conductivity Clamp BT-110. All the samples were cut into stripes (30 × 5 mm). The conductivities of acid-form membranes were measured in DI water at varied temperatures ranging from 25 to 80°C. The films were immersed in water for at least 10 min to obtain consistent resistance at each temperature. The resistance of sample was acquired from the slope of a linear voltage-current plot (0.3–0.8 V). The proton conductivity σ (S/cm) was calculated as follows:

(6) $$\begin{array}{l} \sigma =\frac{l}{R\times W\times T} \end{array}$$

where ohmic resistance (*R*) was calculated from the slope of potential-current plot, *l* is the distance between platinum wires (0.425 cm), *W* and *T* are the width and thickness of the membrane, respectively. The average value was recorded from at least 3 samples of each membrane.

## Results and Discussion

### Copolymer Synthesis and Characterization

The synthetic route for iptycene-containing sulfonated copolymers is shown in Scheme 1 via condensation polymerization following nucleophilic aromatic substitution (S_N_Ar) mechanism. Two structure parameters were adjusted in these two series of copolymers: the degree of sulfonation (ranging from 35 to 50%) and the molar content of iptycene unit (ranging from 33 to 66%). This has allowed systematic investigation of the structure-property relationship for these new iptycene-containing sulfonated polysulfones to elucidate the effect of iptycene structure on membrane properties. It should be noted that the molar content of TRP or PENT in the copolymers was limited due to the relatively low reactivity between TRP/PENT and SDCDPS as well as the low solubility of pentiptycene-based polymers, which prevented the formation of polymers with sufficiently high molecular weight for film casting. The chemical structures and the composition of iptycene-containing disulfonated copolymers were confirmed by ^1^H NMR spectroscopy. Figure S1 shows representative ^1^H NMR spectrum of a triptycene-based copolymer and a pentiptycene-based copolymer with peak assignment according to their structures. The actual molar contents of iptycene units in the copolymers were determined by the peak integration ratio of the iptycene moieties and the BP unit. The results matched the target values, indicating successful synthesis of both copolymer series with well-controlled compositions. The intrinsic viscosity values of iptycene-containing copolymers are mostly in the range of 0.50~0.75 dL/g (Table 1), indicating sufficiently high molecular weight for film casting as shown later. The relatively lower IV values of TRP/PENT-BP series compared to the BPSH series indicating lower molecular weight than BPSH series due to lower reactivity of TRP and PENT series caused by the steric hindrance and poor solubility.

**Table 1:** Density, intrinsic viscosity (IV), decomposition temperature (T_d, 5%_), and glass transition temperature (T_g_) of iptycene-containing polysulfone copolymers.

**Copolymers**	**Density (g/cm^**3**^)**	***IV* (dL/g)^**a**^**	***T_***d*,**_*_**5%**_ (^**°**^C)^**b**^**	***T_***g***_* (^**°**^C)^**c**^**
TRP-BP 1:1-35	1.41 ± 0.04	0.48 ± 0.01	383	254
TRP-BP 2:1-35	1.37 ± 0.06	0.54 ± 0.01	355	277
TRP-BP 1:2-40	1.33 ± 0.01	0.70 ± 0.01	392	281
TRP-BP 1:1-40	1.37 ± 0.01	0.52 ± 0.01	404	280
TRP-BP 1:2-50	1.38 ± 0.01	0.52 ± 0.01	382	280
TRP-BP 1:1-50	1.39 ± 0.02	0.42 ± 0.01	388	291
PENT-BP 1:2-40	1.33 ± 0.01	0.62 ± 0.01	427	296
PENT-BP 1:1-40	1.31 ± 0.01	0.75 ± 0.01	394	299, 311
PENT-BP 1:2-50	1.32 ± 0.02	0.50 ± 0.01	392	309
BPSH 30	1.37 ± 0.02	0.82 ± 0.01	352	248
BPSH 40	1.42 ± 0.03	1.16 ± 0.01	382	260
BPSH 50	1.40 ± 0.01	0.62 ± 0.01	341	271

a *Measured on salt-form samples in 0.05 M LiBr NMP solution at 25°C*.

b *5% weight-loss temperature measured on acid-form samples under N_2_ atmosphere*.

c *Measured on acid-form samples by DSC at 10°C/min, N_2_*.

As shown in Table 1, at given degree of sulfonation, the dry membrane densities of iptycene-containing copolymers, both TRP-BP series and PENT-BP series were all lower than BPSH series without iptycene moieties. It seems to suggest that incorporating iptycene-based structure units might effectively disrupt the chain packing generating larger fractional free volume. Additional analysis reveals that the PENT-BP series exhibit lower densities than TRP-BP series at equivalent sulfonation degree (except TRP-BP 1:2-40). This observation could be rationalized by the larger free volume cavities than those of triptycene units, in particular, the internal free volume defined by the clefts between the benzene “blades” of pentiptycene units (Luo et al., 2015).

### Thermal Analysis and Mechanical Properties

**Figure 1:**
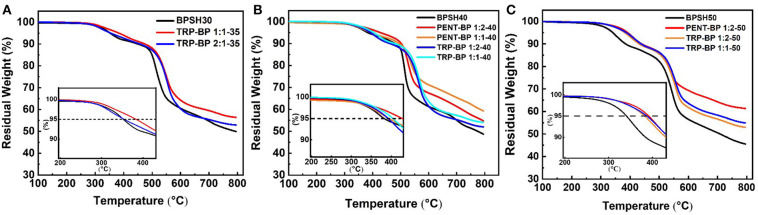
The TGA curves of disulfonated copolymers at comparable sulfonation degrees.

The thermal stability of the acid-form copolymer membranes was studied by thermogravimetric analysis (TGA) in nitrogen flow and the results of 5% weight-loss temperature (T_d, 5%_) are listed in Table 1. All the membranes showed a typical two-step degradation behavior (Figure 1). The first stage weight loss around 350°C was due to the loss of -SO_3_H group (Gong and Zhang, 2011). As such, copolymers with higher degree of sulfonation usually show lower thermal stability. The second stage loss above 500°C was ascribed to the degradation of the polymer main chains. All of the tested polysulfones show *T*_*d*_,_5%_ higher than 300°C, indicating sufficient thermal stability for use in PEMFCs. As a general observation, incorporation of iptycene moieties into the backbone appeared to improve the thermal stability of the copolymers given the same degree of sulfonation, which could be ascribed to the high rigidity of iptycene-based polymer structures and strong intermolecular and intramolecular π-π interactions. For example, at the same degree of sulfonation of 50% (Figure 1C), BPSH 50 has a *T*_*d*_,_5%_ value of 341°C, which is significantly lower than the iptycene-containing copolymers, i.e., PENT-BP 1:2-50 (392°C), TRP-BP 1:2-50 (382°C), and PENT-BP 1:2-50 (388°C). The same trend is observed when comparing the copolymers with similar IEC_w_ values. For example, PENT-BP 1:1-40 (1.27 meq/g) and TRP-BP 1:1-35 (1.27 meq/g) showed *T*_*d*_,_5%_ values of 394 and 383°C, respectively, which are much higher than 352°C of BPSH 30 (1.25 meq/g).

The glass transition temperatures were determined using TA Q2000 and the results are shown in Table 1. It can be seen that within the same copolymer series, *T*_*g*_ increases with increasing the degree of sulfonation. This could be attributed to the fact that higher degree of sulfonation induces stronger intermolecular interactions by ionic effect that stiffens polymer backbone. As expected, iptycene-based sulfonated polysulfones show higher *T*_*g*_ than BPSH series at the equivalent sulfonation degree. For example, the *T*_*g*_ of TRP-BP 1:1(2)-40 and PENT-BP 1:1(2)-40 increased by ~8 and 15%, respectively, compared to BPSH 40. The observed increase in *T*_*g*_ suggests that the bulky and shape-persistent structures of iptycene units effectively increase the polymer chain rigidity. On the other hand, the strong intermolecular and intramolecular π-π interactions between the benzene rings of iptycene units might also contribute to higher glass transition temperatures. This may also explains that PENT-BP series with more fused arene rings exhibit higher *T*_*g*_ than TRP-BP copolymers at similar sulfonation degree. While previous studies reported that two *T*_*g*_'s might be observed for BPSH 50 and BPSH 60 (Wang et al., 2002), here we were able to observe only one *T*_*g*_ for BPSH 50 and other copolymers at 50% sulfonation degree. It should be noted that the degradation of -SO_3_H group starts at ~250°C (Figure 1), which may also obscure the *T*_*g*_ detection. Interestingly, two distinct *T*_*g*_'s (299 and 311°C) were detected for PENT-BP 1:1-40 (Figure S2), which correspond to the *T*_*g*_ of the polymer matrix and ionic clusters, respectively. The unusual phenomenon here might be rationalized by the lower sulfonation degree and excellent thermal stability (*T*_*d*, 5%_ = 394°C) of PENT-BP 1:1-40.

Mechanical properties of membranes were tested on the acid-form films in fully hydrated state at room temperature. Depending on the degree of sulfonation and the content of iptycene units, the copolymer membranes showed tensile strength in a range of 16-48 MPa, Young's modulus of 0.11-0.48 GPa, and elongation at break of 19–75% (Table S1). While other factors, such as molecular weight, may have strong impact on mechanical properties, the iptycene-containing copolymers, especially the pentiptycene series, seem to be stiffer (higher Young's modulus) but less ductile (smaller elongation at break) than the non-iptycene-containing BPSH series due to much higher chain rigidity (higher *T*_*g*_) upon the incorporation of iptycene units.

### Ion Exchange Capacity, Water Uptake, and Swelling Ratio

Ion exchange capacity (IEC_w_, meq/g) is an important factor for the PEM performance: high IEC_w_ leads to high water uptake facilitating the proton transport; however, excessive water uptake causes severe dimensional instability and ion dilution, weakening the mechanical strength and reducing proton conductivity. To achieve high PEMFC performance, it is desirable to optimize the IEC_w_ by tuning the polymer composition to balance mechanical integrity and proton conductivity. The dry-weight IEC_w_ was obtained from the acid-base titration method using phenolphthalein as indicator, and the results were in agreement with the theoretical values calculated from the monomer feed ratio during polymerization (Table 2). The 0.1~0.2 meq/g deviation between calculations and titration results probably came from incomplete ion exchange and experimental errors. As expected, increasing the degree of sulfonation led to increases in IEC_w_ values in all three copolymer series. Given the same degree of sulfonation, iptycene-containing copolymers have lower IEC_w_ values than the BPSH series. This is due to higher molecular weight of repeat units in the iptycene series.

**Table 2:** Ion exchange capacity (IEC), water uptake (WU), and swelling ratio (SR).

**Copolymers**	**${\text{IEC}}_{\text{w}}^{\text{Cal},\text{a}}$ (meq/g)**	**${\text{IEC}}_{\text{w}}^{\text{Mea},\text{b}}$ (meq/g)**	**IEC_**v**_(wet)^**c**^ (meq/cm^**3**^)**	**WU (%)**	**SR (%)**
TRP-BP 1:1-35	1.38	1.27 ± 0.01	1.36 ± 0.07	65 ± 4	32 ± 5
TRP-BP 2:1-35	1.34	1.23 ± 0.01	1.35 ± 0.07	64 ± 3	25 ± 4
TRP-BP 1:2-40	1.61	1.46 ± 0.01	1.22 ± 0.06	67 ± 3	59 ± 7
TRP-BP 1:1-40	1.55	1.43 ± 0.01	1.33 ± 0.08	70 ± 6	47 ± 9
TRP-BP 1:2-50	1.92	1.76 ± 0.02	0.73 ± 0.07	155 ± 5	231 ± 33
TRP-BP 1:1-50	1.88	1.70 ± 0.02	0.63 ± 0.04	258 ± 12	274 ± 23
PENT-BP 1:2-40	1.44	1.35 ± 0.01	1.55 ± 0.08	31 ± 4	16 ± 5
PENT-BP 1:1-40	1.36	1.27 ± 0.01	1.49 ± 0.06	28 ± 5	12 ± 4
PENT-BP 1:2-50	1.75	1.60 ± 0.01	1.29 ± 0.13	99 ± 17	61 ± 16
BPSH 30	1.34	1.25 ± 0.01	1.53 ± 0.05	24 ± 3	12 ± 3
BPSH 40	1.72	1.59 ± 0.02	1.54 ± 0.06	44 ± 2	47 ± 4
BPSH 50	2.0	1.96 ± 0.02	1.41 ± 0.07	115 ± 20	95 ± 10

a *Calculated from monomer feed ratio during polymerization*.

b *Determined from titration method using 0.01 M NaOH solution*.

c *Calculated from Equation (5)*.

**Figure 2:**
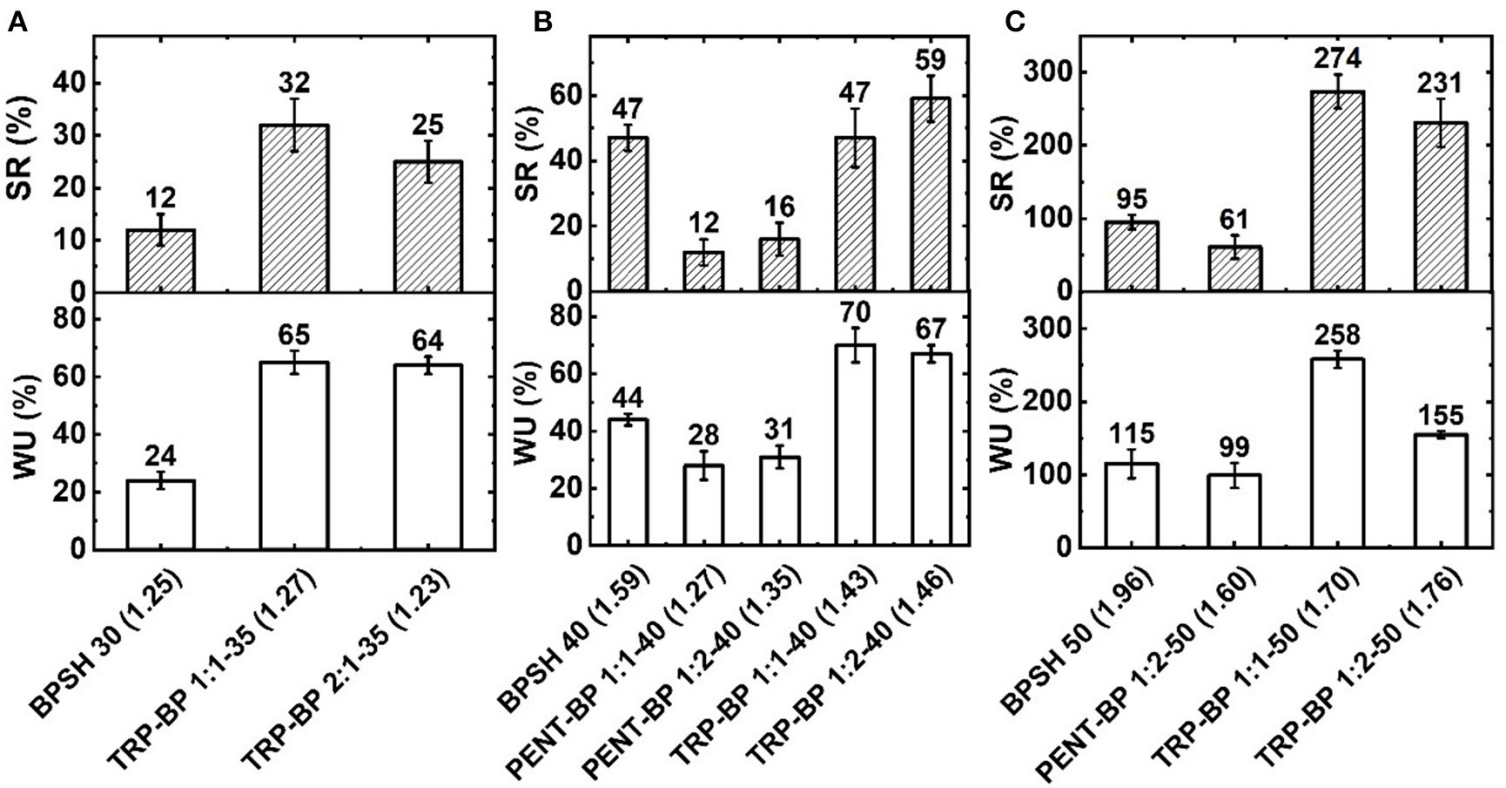
Water uptake (WU) and swelling ratio (SR) for copolymers with **(A)** 30 or 35%, **(B)** 40%, and **(C)** 50% degree of sulfonation at 25°C. The number in the brackets is dry weight-based IEC_w_ (meq/g) determined from titration method.

The water uptake and swelling behavior results are depicted in Figure 2. It is noted that there are multiple interplaying factors that regulate the water uptake and swelling behavior of the copolymer membranes, including IEC_w_, fractional free volume, hydrophobicity, and intra- and intermolecular interactions like π-π stacking. For all three copolymer series, a general trend is observed as expected that: higher IEC_w_ or sulfonation degree yields higher water uptake and swelling ratio. Another general observation (with few exceptions as explained below) is that with similar IEC_w_ or degree of sulfonation, iptycene-containing series seem to have larger water uptake (due to larger fractional free volume) but less volume swelling (possibly due to strong supramolecular interactions) than BPSH series. For example, comparing to BPSH 30 and BPSH 40, TRP-BP 1(2):1-35 and TRP-BP 1:1(2)-40 copolymers with similar or even lower IEC showed much higher water uptake while maintaining dimensional stabilities (Figures 2A,B). Usually water uptake is proportional to free volume according to Yasuda's theory (Yasuda et al., 1969). The increased water uptake suggests that incorporation of bulky triptycene units increases the free volume compared to BPSH series. While the absolute values of swelling ratios for TRP-BP series are higher than those of BPSH series at 35 and 40% sulfonation degrees, TRP-BP series are still deemed to have better dimensional stability considering their much higher water uptake than BPSH series. For example, comparing BPSH 40 and TRP-BP 1:1-40 copolymers, the latter could take nearly 60 wt% more water but had the same volume swelling ratio of 47% as that of BPSH40. This could be attributed to the supramolecular interlocking and possible chain threading interactions between iptycene moieties, in addition to their strong π-π stacking interaction, which effectively suppress the water swelling by transferring strain from one polymer chain to another (Tsui et al., 2007; Gong et al., 2011; Aboki et al., 2018). Moreover, increasing the iptycene molar content in the polymer backbone led to further improved membrane swelling behavior. For example, compared to TRP-BP 1:2-40 membrane containing 33 mol% TRP units, TRP-BP 1:1-40 membrane containing 50 mol% TRP unit has slightly higher water uptake (70 vs. 67 wt%) but much lower swelling ratio (47 vs. 59 vol%). This observation further supports the conclusion that iptycene units are instrumental in suppressing excessive water swelling in ionic polymers.

The trend became more complicated in copolymers involving pentiptycene units and very high sulfonation degree. Despite of the possible higher free volume suggested by the lower dry polymer densities, the PENT-BP copolymers with 40% sulfonation degree didn't show high water uptake as expected. This might be ascribed to their low IEC_w_ values and the more hydrophobic characteristic caused by excessive π-π interactions and highly bulky structures of pentiptycene units, which may block the penetration of water molecules especially at low sulfonation degree. However, the same phenomena of suppression of swelling was observed for the PENT-BP copolymers due to the aforementioned strong π-π stacking and supramolecular interactions. At high sulfonation degree (50%), all the membranes showed markedly increased water uptake and swelling ratio as Figure 2C shows. In particular, PENT-BP 1:2-50 maintained excellent water swelling management with a very high water uptake (99 wt%) but a moderate swelling ratio of 61%. The excellent water uptake and swelling resistance might be explained by the synergistic effects of high fractional free volume, strong intra- and inter-molecular interactions between pentiptycene moieties, and high polymer backbone rigidity. However, excessive swelling behavior was observed for TRP-BP 1:1-50 and TRP-BP 1:2-50 copolymer membranes. It seems that when water uptake surpasses certain critical point, the absorbed water may serve as plasticizer and breaks the inter-chain association formed through supramolecular interlocking and chain threading, resulting excessive swelling behavior. Additionally, TRP-BP 1:1(2)-50 copolymers had relatively low molecular weights (as evidenced by their low intrinsic viscosities) and thus might fail to form stable supramolecularly interlocked structures as in other copolymers. For instance, TRP-BP 1:1-50 had the lowest intrinsic viscosity (0.42) among all the copolymers, which consequently showed the highest water uptake and swelling ratio.

### Oxidative Stability

**Figure 3:**
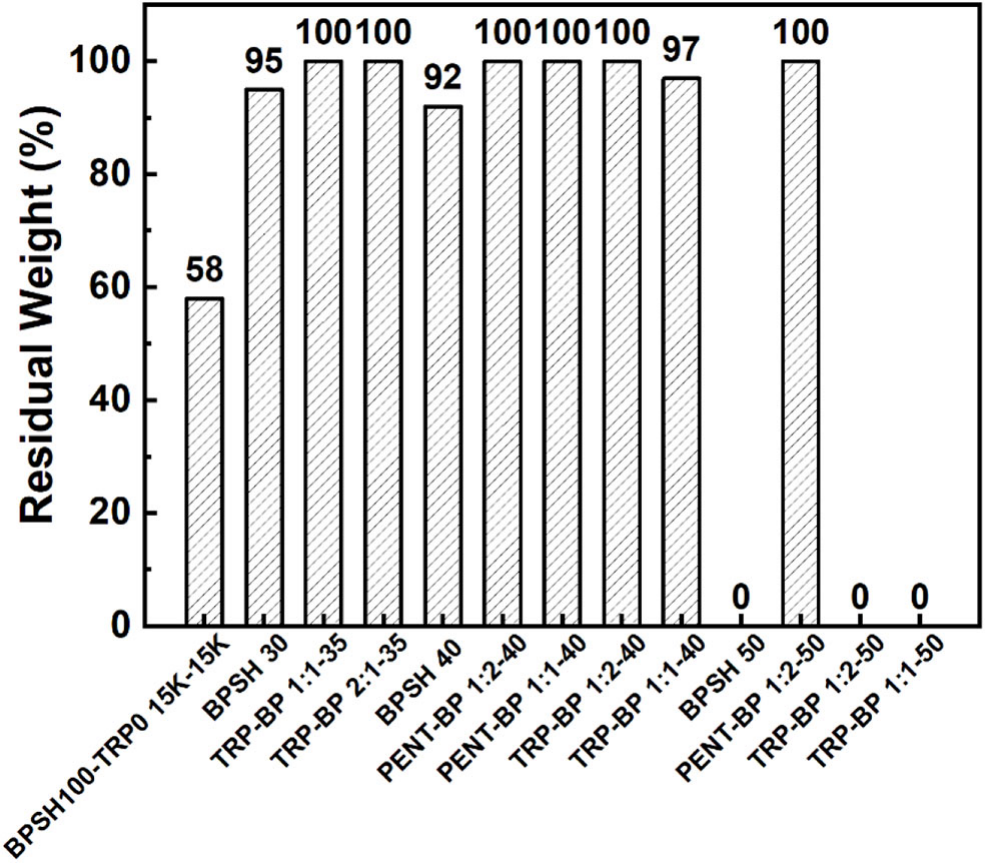
Comparisons of oxidative stabilities of iptycene-based copolymers and BPSH copolymers. BPSH100-TRP0 15K-15K is a triptycene-based multiblock copolymer reported in our previous work (Aboki et al., 2018).

In PEMFC operation, incomplete reduction reaction along with oxygen diffusion through membrane would generate ·OH and ·OOH radicals, which are responsible for accelerating the degradation of PEM membranes (Wang and Capuano, 1998; Hübner and Roduner, 1999; Panchenko et al., 2004; Gong and Zhang, 2011). It is believed that the attack of hydroxyl radicals mainly occurs on aromatic rings, especially in the *ortho* position to alkyl- and alkyl ether-substituents (Hübner and Roduner, 1999). Hot Fenton's reagent (80°C) is an effective tool that has been frequently used to evaluate the oxidative stability of PEMs. The oxidative stability was quantified by the residual weight percentage after soaking in hot Fenton's reagent for 1 h (Figure 3). It has been shown that the oxidative stability decreases as sulfonation degree increases (Liu et al., 2007) as BPSH series exhibited. At higher sulfonation degree, BPSH 50 and TRP-BP1:1(2)-50 either dissolved or broken into small pieces during the tests. This could be attributed to the open membrane structure due to excessive water uptake and swelling are more vulnerable to radical attack. It is worth noticing that PENT-BP 1:2-50 showed excellent chemical stability without detectable weight loss in the test, which might be related with the relatively low IEC_w_ and excellent dimensional stability as a result of strong inter-chain association induced by π-π stacking and supramolecular interaction. At lower sulfonation degrees, all the sulfonated random polysulfone copolymers exhibit good oxidative stabilities with residual weight over 90%. In comparison, iptycene-based copolymers all exhibited higher oxidative stabilities than their BPSH counterparts. The excellent oxidative stabilities could be attributed to the strong supramolecular interlocked structures and π-π interactions induced by iptycene units, which shield the polymer backbones from the radical attack.

### Proton Conductivity

**Figure 4:**
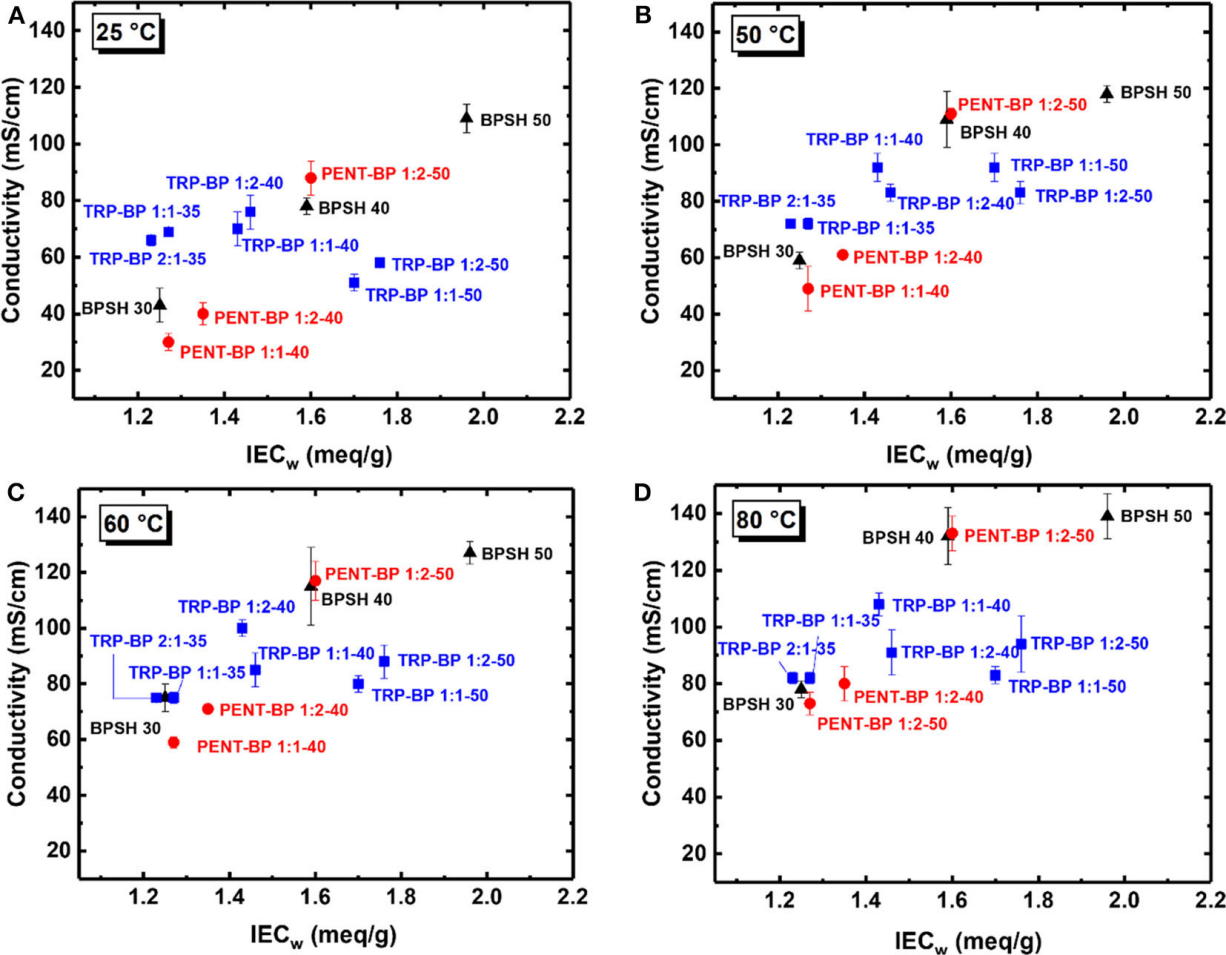
Proton conductivities of BPSH, TRP-BP, and PENT-BP copolymer series at **(A)** 25°C, **(B)** 50°C, **(C)** 60°C, and **(D)** 80°C.

The in-plane proton conductivities of all the membranes were measured at varied temperatures (25–80°C) in deionized (DI) water as tabulated in Table 3. As a general observation, within each copolymer series, increasing IEC_w_ or sulfonation degree led to increased proton conductivity unless excessive water swelling [or very low IEC_v_(wet) values] was involved such as TRP-BP 1:1(2)-50 series. Across the three copolymer series, there are mixed trends comparing the iptycene-containing systems with the BPSH series. To examine the fundamental structure-property relationship, the proton conductivities at varied temperatures were plotted as a function of dry-weight IEC_w_ determined from titration method as Figure 4 shows. For BPSH and PENT-BP series, it can be seen that proton conductivities increased almost linearly with increasing IEC_w_ under all four temperatures, while TRP-BP series showed a different trend, particularly, for the ones with 50% sulfonation degree whose relatively low molecular weights and excessive swelling might contribute to this unusual trend. PENT-BP 1:1-40 and PENT-BP 1:2-40 showed lower conductivities than BPSH 30 with similar IEC_w_, consistent with the low water uptake caused by higher hydrophobicity and excessive π-π interactions. The much higher water uptake of PENT-BP 1:2-50 led to higher proton conductivities than BPSH 40 even they share similar dry weight IEC_w_ (1.60 meq/sg and 1.59 meq/g, respectively). Interestingly, PENT-BP 1:2-50 with much higher water uptake than BPSH 40 (99 vs. 44%) showed much improved conductivities at 25°C (88 vs. 78 mS/cm) but similar conductivities of ~132 mS/cm at higher temperatures. This phenomenon could be explained by the proton transport mechanism: the translational mode gradually transits from Grotthuss mechanism to vehicle-type mechanism with increasing temperature (Kreuer, 1996). Different from the “smooth” backbone of BPSH 40, the bulky structure and pendant fused arene rings of pentiptycene units in PENT-BP series increase the pathway tortuosity that might slow down the proton transporting vehicles (i.e., H_3_O^+^), compromising the conductivity increase at high temperatures.

**Table 3:** Proton conductivity as a function of temperature and calculated activation energy (*E*_*a*_).

**Copolymers**	**IEC_**w**_(dry) (meq/g)**	**IEC_**v**_(wet)^**a**^ (meq/cm^**3**^)**	**Proton conductivity (mS/cm)**				***E_***a***_*^**b**^ (kJ/mol)**
			**25^**°**^C**	**50^**°**^C**	**60^**°**^C**	**80^**°**^C**	
TRP-BP 1:1-35	1.27 ± 0.01	1.36 ± 0.07	69 ± 1	72 ± 2	75 ± 2	82 ± 2	2.68
TRP-BP 2:1-35	1.23 ± 0.01	1.35 ± 0.07	66 ± 2	72 ± 1	75 ± 1	82 ± 2	3.41
TRP-BP 1:2-40	1.46 ± 0.01	1.22 ± 0.06	76 ± 6	83 ± 3	85 ± 6	91 ± 8	3.37
TRP-BP 1:1-40	1.43 ± 0.01	1.33 ± 0.08	70 ± 6	92 ± 5	100 ± 3	108 ± 4	7.68
TRP-BP 1:2-50	1.76 ± 0.02	0.73 ± 0.07	58 ± 1	83 ± 4	88 ± 6	94 ± 10	7.88
TRP-BP 1:1-50	1.70 ± 0.02	0.63 ± 0.04	51 ± 3	72 ± 2	80 ± 3	83 ± 3	8.81
PENT-BP 1:2-40	1.35 ± 0.01	1.55 ± 0.08	40 ± 4	61 ± 1	71 ± 1	80 ± 6	11.4
PENT-BP 1:1-40	1.27 ± 0.01	1.49 ± 0.06	30 ± 3	49 ± 8	59 ± 2	73 ± 4	14.4
PENT-BP 1:2-50	1.60 ± 0.01	1.29 ± 0.13	88 ± 6	111 ± 2	117 ± 7	133 ± 6	6.56
BPSH 30	1.25 ± 0.01	1.53 ± 0.05	43 ± 6	59 ± 3	75 ± 5	78 ± 3	10.1
BPSH 40	1.59 ± 0.02	1.54 ± 0.06	78 ± 3	109 ± 10	115 ± 14	132 ± 10	8.36
BPSH 50	1.96 ± 0.02	1.41 ± 0.07	109 ± 5	118 ± 3	127 ± 4	139 ± 8	3.88

a *Calculated using Equation (5)*.

b *Calculated using Equation (7)*.

For TRP-BP series, the situations were more complicated. TRP-BP 1:1-35 and TRP-BP 2:1-35 are more conductive at all the temperatures than BPSH 30, PENT-BP 1:1-40 and PENT-BP 1:2-40, which had similar IEC_w_ values. For example, at 25°C, TRP-BP 2:1-35 and TRP-BP 1:1-35 have proton conductivities of 66 and 69 mS/cm, respectively, much higher than BPSH 30 (43 mS/cm), PENT-BP 1:1-40 (30 mS/cm), and PENT-BP 1:2-40 (40 mS/cm). The excellent proton conductivities of TRP-BP series even with low IEC_w_ values are consistent with their high water uptake of ~65% (Figure 2). As for the effect of iptycene content, the proton conductivities of TRP-BP 1:1-40 (*IEC*_*w*_ = 1.43 meq/g) surpassed TRP-BP 1:2-40 (1.46 meq/g) at 50°C and finally reached 108 mS/cm at 80°C (cf. Table 3 or Figure 4), approximately 16% greater than the latter. The conductivity increase at higher temperatures is possibly due to more stable interlocked structures in the copolymer with higher iptycene content: higher percentage of triptycene units might retain the connectivity of ionic domains facilitating the vehicle-type proton transport at high temperatures. However, TRP-BP 1:1-50 and TRP-BP 1:2-50 ionomers with high IEC_w_ values suffered cliff-like drops in proton conductivities, consistent with the excessive water uptake and water-swollen behaviors, where the ion concentration is too dilute to form continuous conduction. As such, TRP-BP 1:1-50 and TRP-BP 1:2-50 with very low volume-based IEC_v_(wet) values of ~ 0.7 meq/cm^3^ exhibited lower conductivities than BPSH 40, PENT-BP 1:2-50, and BPSH 50, which have much higher IEC_v_(wet) values (≥1.29 meq/cm^3^). As mentioned earlier, the excessive water-swollen stress may override the π-π interactions and deteriorate the interlocked structures from triptycene units.

**Figure 5:**
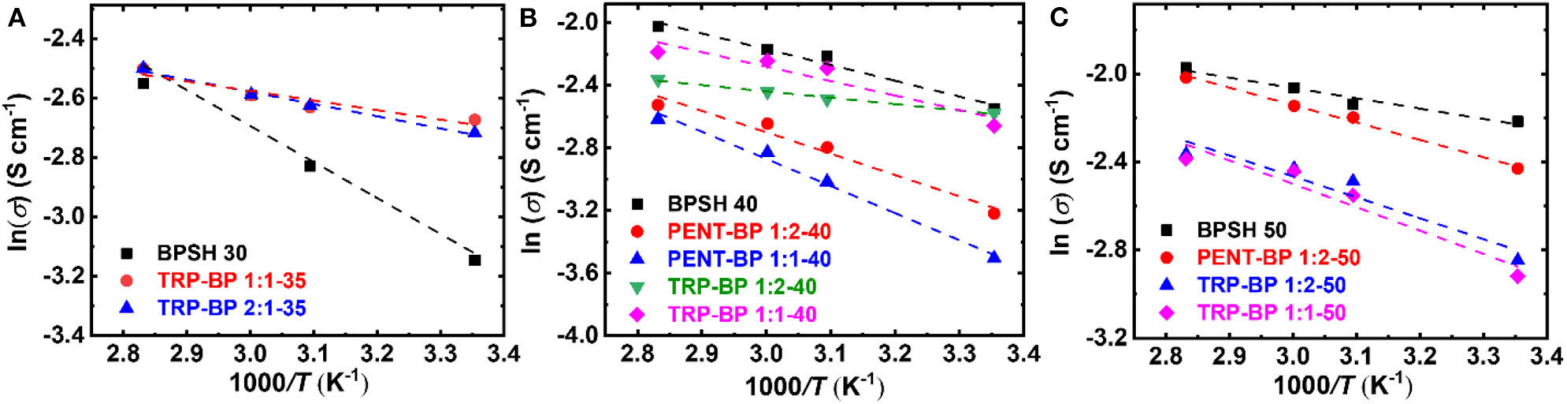
Arrhenius plot of log conductivity vs. 1*/T* for copolymers with **(A)** 30 or 35%, **(B)** 40%, and **(C)** 50% degree of sulfonation.

The relationship between the proton conductivity (σ) and temperature (*T*) was well-expressed by the Arrhenius equation as follows (Lee et al., 2005),

(7) $$\begin{array}{l} \ln\sigma =\ln\sigma_{0}-\frac{E_{a}}{RT} \end{array}$$

where σ_0_ is the frequency factor, *R* is gas constant (8.314 J mol^−1^ K^−1^), and *T* is absolute temperature (K). The activation energy (*E*_*a*_) was calculated from the slope of linear fit of ln σ vs. 1/*T* as Figure 5 shows and the calculated values were tabulated in Table 3. It should be noted that the activation energy values could be affected by multiple non-structure related factors such as film-casting conditions, testing method, etc. Although some samples tested here didn't rigorously follow the Arrhenius behavior (such as BPSH 30), most copolymers show rather linear correlation between *ln* σ and 1/*T* with linearity approaching unity. Generally, a high activation energy value means proton conductivity is more sensitive to temperature change. As demonstrated in previously studies (Kim et al., 2003), the activation energy of BPSH series decreased as sulfonation degree increased due to the formation of continuous ionic domains (cf. Table 3). For PENT-BP series, the activation energy is higher than BPSH at equivalent sulfonation degree. For example, the activation energy of PENT-BP 1:1-40 (14.39 kJ/mol) is ~27 and ~72% higher than PENT-BP 1:2-40 and BPSH 40. This phenomenon could be attributed to that the dynamic ion transport gradually outweighs the excessive π-π interactions as temperature increases. As such, the proton conductivity of PENT-BP changes more rapidly with temperatures. For TRP-BP series, there is no obvious trend of activation energy as observed in PENT-BP and BPSH series.

## Conclusion

Disulfonated iptycene-based poly(arylene ether sulfone) random copolymers, i.e., TRP-BP and PENT-BP, were synthesized via direct copolymerization of disulfonated monomer. The sulfonation degree and iptycene contents were systematically varied to investigate the effects of intrinsic free volume and IEC on proton exchange membrane performance. Due to the combined effects of high free volume, supramolecularly interlocked structure, and strong π-π interaction associated with triptycene moieties, TRP-BP copolymers showed high water uptake with low swelling ratio as compared to BPSH series. Correspondingly, they exhibited much higher proton conductivities from 25 to 80°C than BPSH counterparts with similar IEC_w_ values. However, with even higher intrinsic free volume, pentiptycene-based PENT-BP series didn't show much improved proton conductivities at low degree of sulfonation, possibly due to their higher hydrophobicity (evidenced by low water uptake and swelling ratio) that compromises IEC and thus proton conductivity. For PENT-BP 1:2-50, its high sulfonation degree might overcome the hydrophobic characteristic, resulting in high water uptake, good dimensional stability as well as high conductivity. The TRP-BP and PENT-BP copolymer membranes exhibited much improved oxidative stability after 1 h hot Fenton's reagent treatment than BPSH series, which could be attributed to the strong supramolecular interlocked structures and π-π interactions induced by iptycene units, which shield the polymer backbones from the attack of ·OH and ·OOH radicals.

## Data Availability Statement

All datasets presented in this study are included in the article/Supplementary Material.

## Author Contributions

TW, JA, and RG conceived the idea and designed the experiments. TW, TL, and JA synthesized the membranes. TW performed most of the characterization and measurement. TW and RG analyzed the data and wrote the report. All authors read and approved the final manuscript.

## Conflict of Interest

The authors declare that the research was conducted in the absence of any commercial or financial relationships that could be construed as a potential conflict of interest.
