# Supplementary material for: Disulfonated Poly(arylene ether sulfone) Random Copolymers Containing Hierarchical Iptycene Units for Proton Exchange Membranes
Source: Front Chem. 2020 Aug 4;8:674. doi: 10.3389/fchem.2020.00674 (PMC7417612; doi:10.3389/fchem.2020.00674)
Supplement: Supplementary file 1 [file Data_Sheet_1.PDF]

Supplementary Materials

# Disulfonated Poly(arylene ether sulfone) Random Copolymers Containing Hierarchical Iptycene Units for Proton Exchange Membranes

**Tao Wang, Tianyun Li, Joseph Aboki, Ruilan Guo***

Department of Chemical and Biomolecular Engineering, University of Notre Dame, Notre Dame, IN, USA

***Correspondence:**
Professor Ruilan Guo
rguo@nd.edu

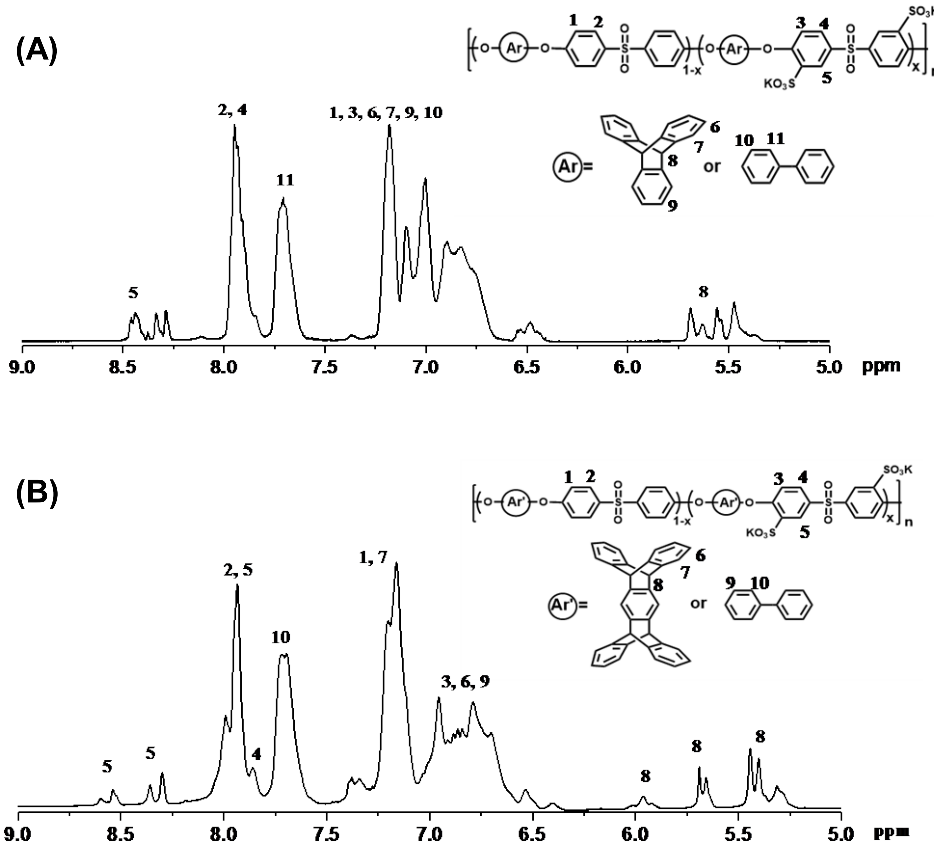


**Supplementary Figure S1.** $^{1}$H NMR spectra of (A) TRP-BP 1:1-35 and (B) PENT-BP 1:2-40

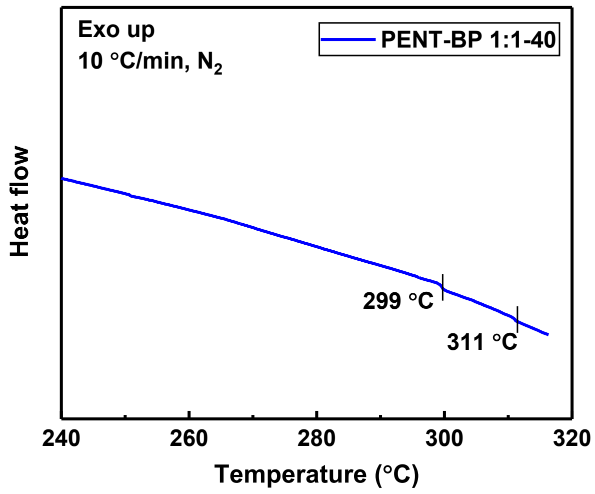


**Supplementary Figure S2.** DSC thermogram of PENT-BP 1:1-40.

**Supplementary Table S1.** Mechanical properties of representative acid-form PENT-BP, TRP-BP and BPSH copolymer membranes.

| | **Tensile strength (Mpa)** | **Elongation at break (%)** | **Young's modulus (Mpa)** |
|---|---|---|---|
| BPSH 30 | 56 ± 2 | 80 ± 16 | 350 ± 29 |
| BPSH40 | 42 ± 3 | 90 ± 21 | 120 ± 7 |
| PENT-BP 1:2-40 | 36 ± 1 | 30 ± 6 | 484 ± 7 |
| PENT-BP 1:1-40 | 36 ± 1 | 19 ± 2 | 480 ± 30 |
| PENT-BP 1:2-50 | 16 ± 3 | 35 ± 3 | 110 ± 13 |
| TRP-BP 1:1-35 | 47 ± 5 | 75 ± 16 | 280 ± 30 |
| TRP-BP 2:1-35 | 32 ± 4 | 44 ± 8 | 200 ± 21 |
